# Cholesterol Promotes Interaction of the Protein CLIC1 with Phospholipid Monolayers at the Air–Water Interface

**DOI:** 10.3390/membranes6010015

**Published:** 2016-02-11

**Authors:** Khondker R. Hossain, Heba Al Khamici, Stephen A. Holt, Stella M. Valenzuela

**Affiliations:** 1 School of Life Sciences, University of Technology Sydney, Sydney, New South Wales 2007, Australia; khondker.r.hossain@student.uts.edu.au (K.R.H.); Heba.alkhamici@uts.edu.au (H.A.K.); 2 Bragg Institute, Australian Nuclear Science and Technology Organisation (ANSTO), New South Wales 2234, Australia; sph@ansto.gov.au

**Keywords:** CLIC1, membrane insertion, cholesterol, Langmuir monolayer film, phospholipids, POPC, POPE, POPS

## Abstract

CLIC1 is a Chloride Intracellular Ion Channel protein that exists either in a soluble state in the cytoplasm or as a membrane bound protein. Members of the CLIC family are largely soluble proteins that possess the intriguing property of spontaneous insertion into phospholipid bilayers to form integral membrane ion channels. The regulatory role of cholesterol in the ion-channel activity of CLIC1 in tethered lipid bilayers was previously assessed using impedance spectroscopy. Here we extend this investigation by evaluating the influence of cholesterol on the spontaneous membrane insertion of CLIC1 into Langmuir film monolayers prepared using 1-palmitoyl-2-oleoylphosphatidylcholine, 1-palmitoyl-2-oleoyl-sn-glycero-3-phospho-ethanolamine and 1-palmitoyl-2-oleoyl-*sn*-glycero-3-phospho-L-serine alone or in combination with cholesterol. The spontaneous membrane insertion of CLIC1 was shown to be dependent on the presence of cholesterol in the membrane. Furthermore, pre-incubation of CLIC1 with cholesterol prior to its addition to the Langmuir film, showed no membrane insertion even in monolayers containing cholesterol, suggesting the formation of a CLIC1-cholesterol pre-complex. Our results therefore suggest that CLIC1 membrane interaction involves CLIC1 binding to cholesterol located in the membrane for its initial docking followed by insertion. Subsequent structural rearrangements of the protein would likely also be required along with oligomerisation to form functional ion channels.

## 1. Introduction

Cholesterol is an essential constituent of the plasma membrane of most eukaryotic cells, where it is distributed non-randomly and plays a crucial role in membrane organization, dynamics, function and sorting [1,2,3]. Beyond its well documented effects on the physical state of the phospholipid bilayer, cholesterol has been reported to be necessary for the functional activity of many membrane proteins [4,5]. The crucial role of cholesterol in facilitating structural rearrangements of proteins upon association with the lipid bilayer, resulting in the spontaneous conversion of the protein from water-soluble to membrane-bound form, has recently been well documented. This cholesterol-dependent membrane insertion is a widely observed phenomenon and occurs in many bacterial pore-forming toxins (PFTs), specifically the cholesterol-dependent cytolysins (CDCs), as well as the human protein perforin, members of the complement membrane attack complex [4] and the amyloid precursor protein [6,7]. Recent studies with impedance spectroscopy on tethered bilayer membranes (tBLMs) have revealed that cholesterol also regulates the ion channel activity of the eukaryotic chloride intracellular channel protein 1 (CLIC1) into model membranes [8]. 

Chloride intracellular channel proteins (CLICs 1–6) are a family of ubiquitously expressed ion channel proteins that exist either in a soluble state in the cytoplasm or bound to membranes [9,10], where they perform an array of biological functions [11]. In addition to ion channel activity [8,12,13,14], CLIC proteins are involved in cell cycle regulation [15]; neurotransmission [16]; and recently CLICs 1, 2 and 4 were shown to have oxidoreductase enzymatic activity [17]. CLICs contain no known membrane-targeting signal sequences and lack classical membrane spanning domains, but still possess the intriguing property of spontaneous insertion into phospholipid bilayers to form integral membrane spanning channels [9,10,12]. Furthermore, CLIC1 has been categorized as a metamorphic protein because of its ability to reversibly interconvert between two distinct conformational structures within the cytosol labeled as its oxidized or reduced state [18,19]. While the process that governs CLIC1’s membrane interactions and conformational change is still not well understood, it is believed to be influenced by redox environment [19,20,21], the difference in pH between the cytosol (~7.4) and the vicinity of the membrane surface (~5.5) [13,22], and the lipid composition of the membrane [8,12,21]. 

Littler *et al.* (19) showed that upon oxidation, monomeric CLIC1 undergoes a reversible structural transition of its N-terminal domain, exposing a large hydrophobic surface which is stabilized *in vitro* by dimerization [19]. Electrophysiological studies showed that in the presence of a lipid bilayer the oxidized CLIC1 monomer will dock to the membrane, whereas in the absence of lipids it forms a dimer [19]. Controversially, an increase in ion channel activity has also been detected with an increase in the reducing agent dithiothreitol (DTT) [21]. This could suggest that oxidizing conditions lead to a closure of the CLIC1 pore, while reducing conditions result in channel opening and conductance [19,20,21]. In addition, urea-induced equilibrium unfolding studies on reduced soluble CLIC1, in the absence of membranes, revealed that the conformational stability of CLIC1 is pH-dependent [13]. The pH-dependent spontaneous membrane insertion has also been observed for vertebrate CLIC2 and CLIC4 and invertebrate CLICs (EXC-4 and *Dm*CLIC) in which the channel activity was highest at low pH (5.5) and found to decrease with an increase in pH [23,24,25]. However, pH alone does not seem to trigger structural changes in CLIC1 to induce membrane insertion and this was verified by amide hydrogen-deuterium exchange mass spectrometry studies which confirmed that the structure of CLIC1 is unchanged at pH 5.5 when compared to pH 7 [22,26]. Hence, it was speculated that additional factors may be involved in facilitating the conformational changes required for spontaneous membrane insertion of the soluble CLIC1.

Despite differences in sequence and function, structural analysis shows that there is a striking similarity between the human CLICs and many PFTs. The PFTs have two hydrophobic helices that form a helical hairpin in the centre of a bundle, surrounded by six to seven amphipathic α-helices which shield the helical hairpin [4,27,28]. Therefore, it is postulated that in the presence of lipid membranes, these proteins undergo a structural reorganization in order to expose the hydrophobic helices for penetration into the membrane. Correspondingly, the N terminal region of CLIC1 consists of alpha helical bundles of sufficient hydrophobicity, allowing for parallels to be drawn between these proteins. Furthermore, CLIC1 may share a membrane insertion process that is cholesterol-dependent as is known for the CDCs. Recently, studies have also shown that different combinations of phospholipids and cholesterol results in regulation of the functional activity of CLIC1. One of these studies demonstrated that CLIC1 shows significantly greater ion channel activity in membranes containing cholesterol [8]. Hence, it was hypothesized that cholesterol may regulate the spontaneous membrane insertion of CLIC1 followed by oligomerisation of the protein to form functional ion channels in cell membranes [8]. In order to determine the process by which membrane cholesterol regulates the auto-insertion of CLIC1, we here report Langmuir film experiments performed on different combinations of phospholipids and phospholipid-cholesterol monolayers, before and after exposure to CLIC1 protein.

## 2. Results and Discussion

### 2.1. Surface Activity of CLIC1 Protein:

**Figure 1:**
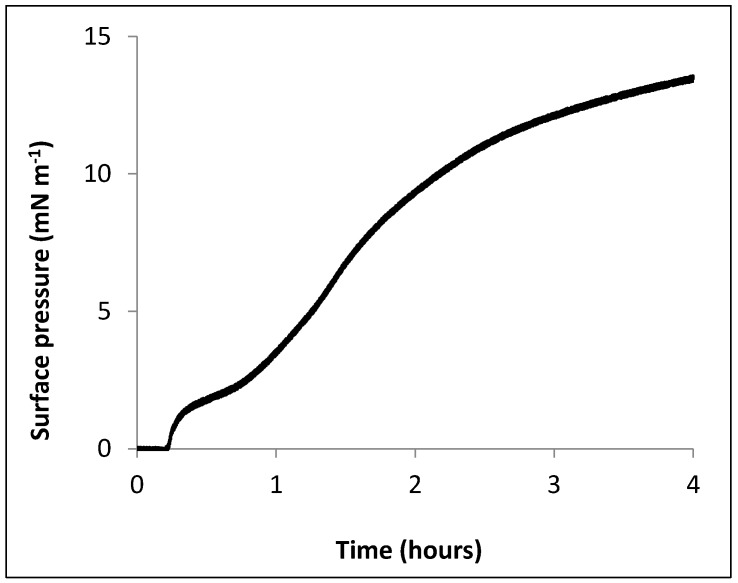
Adsorption isotherm of CLIC1 to an air–water interface at 25 °C and final concentration of 2 µg·mL^−1^ (number of experiments, *n* = 3).

With the aim of understanding CLIC1 interactions with lipid monolayers formed on a buffer subphase, we first assessed CLIC1’s inherent propensity to localize to the air–water interface by measuring changes in surface pressure (π) while maintaining a constant surface area following the injection of protein into the buffer subphase. The increase in surface pressure due to the adsorption of CLIC1 to the air–water interface from the KCl/Hepes buffer subphase (pH 6.5) was measured experimentally using a Langmuir trough. As shown by the adsorption isotherm in Figure 1, after a lag time of 10 min following protein injection into the subphase, CLIC1 rapidly adsorbed to the air–water interface and reached a steady state surface pressure of 14 mN·m^−1^. Remarkably, the adsorption isotherm showed a two-stage behavior where π increased sharply to about 2.5 mN·m^−1^ followed by a plateau and a subsequent slower increase to achieve a final π of 14 mN·m^−1^. This multi-phase adsorption behavior of CLIC1 protein was reproducible and may reflect a protein specific effect following its injection into the buffer subphase. As mentioned previously, the N-terminal domain of soluble CLIC1 has sufficient hydrophobicity to render the molecule amphipathic [9,19]. It appears that CLIC1 has a reasonable affinity for the air–buffer interface, thus the initial sharp increase in π (Figure 1). Strikingly, the adsorption isotherm of CLIC1 is comparable to that observed for the amphipathic Aβ peptide [29]. Furthermore, previously published data of tip-dip single channel electrophysiological studies have described two different ionic conductance states when CLIC1 was added to pure PC lipid bilayers [13]. It was shown that CLIC1, after a variable lag period of null events, first produced small conductance channels with slow kinetics (SCSK) and then underwent a transition to form high conductance channels with fast kinetics (4 times greater than that of SCSK) [13]. Similarly, the ion channel activity of CLIC1 in tBLMs measured using impedance spectroscopy also suggests the existence of two types of protein–membrane interactions [8]. The ability of CLIC1 to conduct in the absence of cholesterol in tBLMs suggests distinct cholesterol-dependent and independent events [8]. The two-stage adsorption behavior of CLIC1 at the air–water interface in the absence of lipids, suggests the protein undergoes phase transitions, which in the presence of a lipid bilayer, would likely involve protein structural rearrangements to form functional ion channels. However such an interpretation must be tempered by the artificial nature of the monolayer system employed in this study.

### 2.2. Interaction of CLIC1 with Phospholipid Monolayer

Due to the complexity of interacting molecules in a cell, we chose to use a reconstituted membrane system to investigate the interaction of CLIC1 protein with lipid films. Previously we have published studies of CLIC1 ion channel activity within tethered lipid bilayers (tBLMs) monitored using impedance spectroscopy [8]. These studies have revealed that the probability of CLIC1 forming functional ion channels when added to membranes lacking cholesterol is very low, compared to adding protein to cholesterol containing membranes [8]. Unlike the tBLMs system, which provides a direct functional measurement of the conductive properties of the protein once embedded within the membrane, the Langmuir film system provides a measure of the protein’s association and/or insertion into the phospholipid film. This has allowed us to independently study and compare these two distinct processes. On the one hand the protein’s functional membrane activity, and on the other, it’s association with, and / or insertion into, the phospholipid monolayer. 

**Figure 2:**
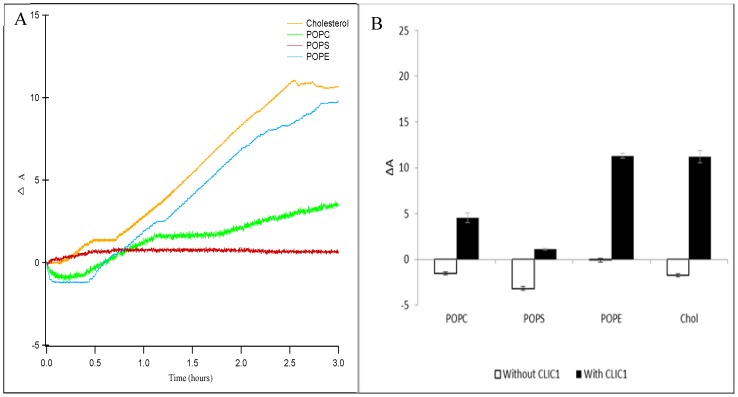
**(A)** Percent area expansion profiles for CLIC1 insertion into cholesterol (orange line), POPC (green line), POPS (red line) and POPE (blue line) monolayers on buffer subphase. **(B)** Corresponding percent area expansion profiles of different lipids or cholesterol monolayers without (white bars) and with (black bars) recombinant CLIC1 wild-type protein after 3 h following protein injection into the subphase. The insertion of CLIC1 in the different lipid or sterol monolayers shows two-stages of interaction, an initial sharp increase in ΔA after a lag phase followed by a plateau and subsequent slow increase in ΔA over a period of 3 h. (*n* = 3)

Langmuir experiments were performed using 1-palmitoyl-2-oleoylphosphatidylcholine (POPC), 1-palmitoyl-2-oleoyl-sn-glycero-3-phospho-ethanolamine (POPE) and 1-palmitoyl-2-oleoyl-*sn*-glycero-3-phospho-L-serine (POPS) lipids alone or in the presence of cholesterol (Chol). Data presented as the percent trough area expansion (ΔA) following CLIC1 insertion into lipid monolayers over a period of 3 h are shown in Figure 2A,B respectively. As seen in Figure 2A, the insertion of CLIC1 protein in the different lipid or sterol monolayers held at a constant pressure of 20 mN·m^−1^ also shows a two-stage interaction behaviour. There is an initial sharp increase in ΔA after a lag phase followed by a plateau and subsequent slower increase in ΔA over a period of 3 h, as previously seen in Figure 1. One may speculate that during the lag phase CLIC1 undergoes a structural change likely to involve a rapid rearrangement of the N-terminal domain for subsequent insertion into the monolayer, followed then by its oligomerisation to form fully functional ion channels. It should also be noted that the surface activity of CLIC1 in the absence of lipids resulted in an increase in surface pressure of only 14 mN·m^−1^, even after 4 h. This is less than the pressure at which the lipid monolayers were held (20 mN·m^−1^) and hence, the area increase is a specific interaction and not as a result of CLIC1’s adsorption to the air–water interface.

In the absence of CLIC1 protein, the lipid monolayers held at 20 mN·m^−1^ (Figure 2B, white bars) were relatively stable over a number of hours followed by an insignificant amount of relaxation or decrease in film area. These minor changes are likely caused by the loss of water from the subphase due to evaporation over time. The percent change in area of the lipid or cholesterol monolayers after 3 h following addition of CLIC1 into the subphase were plotted in Figure 2B (solid black bars). CLIC1 protein favourably interacted with zwitterionic lipid monolayers showing the greatest percentage of surface area expansion (ΔA) of 11.3 ± 0.3% for POPE monolayer followed by a ΔA of 3.7 ± 0.6% for POPC monolayer. CLIC1 protein showed little to no obvious interaction with the anionic POPS monolayer. 

The apparent preference for CLIC1 insertion into the zwitterionic POPE monolayer may be related to the following differences between the three lipids: (i) POPE has the ability to form hydrogen bonds with water and other molecules while POPC cannot [30,31,32] and (ii) that the ethanolamine group is smaller than the choline or serine group and upon compression the lipid molecules may be arranged in a more favourable orientation in the monolayer to facilitate greater protein interaction [31,32,33]. A preferably strong and favourable interaction also exists between the protein and cholesterol molecule and this is evident from the significant amount of CLIC1 insertion (ΔA ~ 11.2%) (Figure 2B) into the cholesterol monolayer. Similar *in vivo* and *in vitro* studies using several different PFTs including listeriolysin O, streptolysin, and pneumolysin have shown that these toxins bind to phospholipid membranes only in the presence of cholesterol and are commonly referred to as cholesterol-dependent cytolysins (CDCs) [4,27,32]. Thus, in order to understand the protein–cholesterol interaction, CLIC1 protein was injected underneath phospholipid monolayers containing cholesterol. 

### 2.3. Interaction of CLIC1 with Phospholipid Monolayer in the Presence of Cholesterol

**Figure 3:**
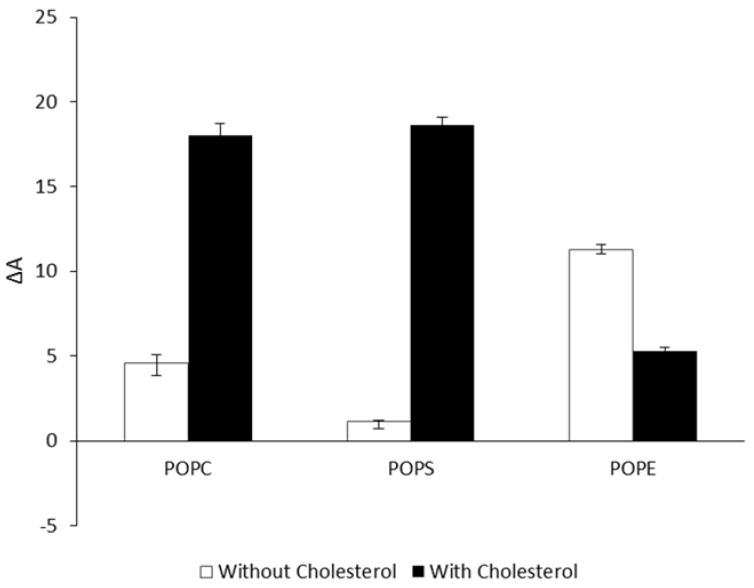
Percent area expansion profiles of different lipid monolayers without (white bars) and with (black bars) cholesterol after 3 h following recombinant CLIC1 protein injection into the subphase. CLIC1 protein showed significant insertion and/or membrane interactions in monolayers containing cholesterol. (*n* = 3)

CLIC1 interactions with phospholipid monolayers were entirely different when cholesterol was included. Comparison of the percent trough area expansion (ΔA) of POPC, POPS and POPE monolayers without cholesterol (Figure 3, white bars) and with cholesterol (Figure 3, black bars) after 3 h following CLIC1 addition to the buffer subphase are shown in Figure 3. The addition of cholesterol to POPC and POPS lead to a significant increase in ΔA of approximately 18.01% for POPC:Chol monolayer and 18.62% (Figure 3) for POPS:Chol monolayer. Hence, this result suggests that the previously reported increase in CLIC1 ion channel conductivity with increasing cholesterol concentration in model membranes (as previously measured by impedance spectroscopy [8]) arises due to an increase in membrane insertion of CLIC1 protein facilitated by membrane cholesterol. In contrast, the ΔA value was reduced from 11.3 ± 0.3% to 5.8 ± 0.24% following addition of CLIC1 in POPE monolayer containing cholesterol (Figure 3). This observation is highly surprising and may possibly be a result of the difference in the packing nature of the lipid/cholesterol monolayer in comparison to that of the pure lipid or cholesterol. However, further analysis is required in order to understand the exact nature of the CLIC1 protein interaction with POPE monolayers but it is clearly evident that the autonomous membrane insertion of CLIC1 is highly regulated by cholesterol and the lipid compositions that are typical of eukaryotic membranes. Previous studies have also suggested that CLIC1 may require specific lipids or lipid combinations to refold or form oligomers after membrane insertion [8,21]. Singh H *et al*. 2006 studied the ion channel activity of CLIC1 protein in planar lipid bilayers and had found that CLIC1 protein failed to form functional ion channels in a variety of lipid mixtures but showed highly reproducible ion channel activity in a POPE, POPS and cholesterol combination of 4:1:1 mole ratio respectively [21]. 

### 2.4. Interaction of CLIC1 with Mixed Lipid Monolayers

**Figure 4:**
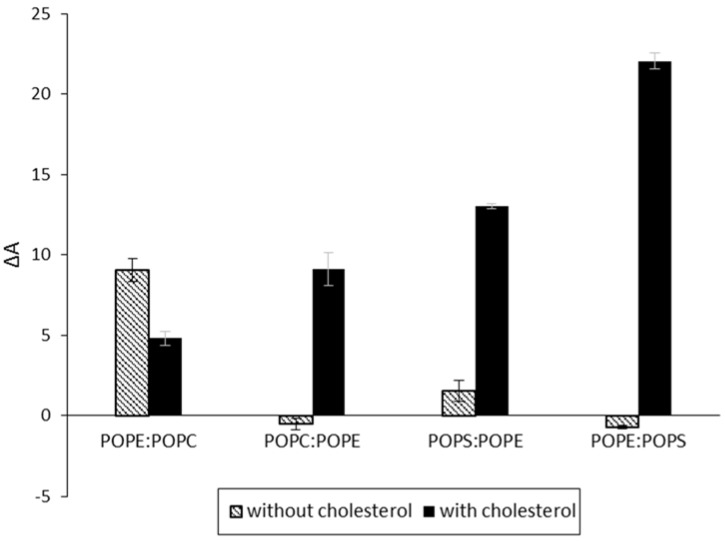
Percent area expansion profiles of monolayers made of different lipid combinations with/without cholesterol (black/white bars) after 3 h of CLIC1 protein injection into the subphase. CLIC1 protein showed significant insertion and/or membrane interactions in POPE:POPS monolayer containing cholesterol. (*n* = 3)

To evaluate the interaction of CLIC1 protein in different lipid environments, a variety of mixed monolayers were formed using a combination of two of the three following lipids: POPC, POPE or POPS in a mole ratio of 5:1 alone or with cholesterol in a mole ratio of 4:1:1. The mixed monolayers were held at a constant pressure of 20 mN·m^−1^ and the percentage surface area expansion ΔA after 3 h of CLIC1 injection into the subphase was recorded (Figure 4). Among the several lipid combinations tested, only the combinations that showed significant CLIC1 protein membrane insertion are shown in Figure 2. CLIC1 protein did not insert into POPE:POPS monolayer but when the monolayer was supplemented with cholesterol, the highest percentage change in area of approximately ~22.04± 0.51% was observed which is consistent with the observations recorded by Singh H. *et al*. 2006 [21]. CLIC1 also showed membrane interaction only after supplementing POPC:POPE and POPS:POPE monolayers with cholesterol. As seen in Figure 2, CLIC1 showed greater insertion in POPE monolayer containing no cholesterol. Similarly, CLIC1 injection into a POPE:POPC (5:1 mole ratio) monolayer resulted in a percentage change in area of ΔA ~9.04 ± 0.71%, whereas adding cholesterol to POPE:POPC monolayer resulted in a reduced ΔA value of 4.8 ± 0.46% (Figure 4).

**Figure 5:**
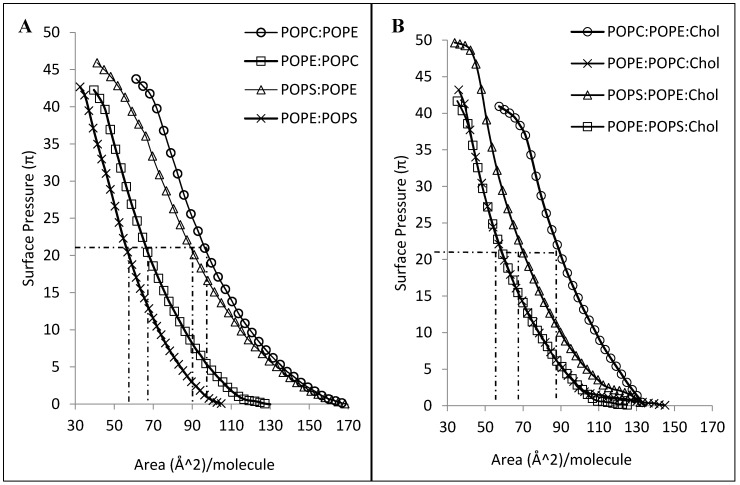
(**A**) Surface pressure-area isotherms of different lipid mixtures of 5:1 mole ratio (**B**) Surface pressure-area isotherms of the same lipid mixtures containing cholesterol in a 4:1:1 mole ratio measured with a Langmuir film balance (*n* = 3).

CLIC1 shows varying degree of interactions with different mixtures of phospholipid monolayers either in the absence or presence of cholesterol. It should be taken into account that although all the phospholipids have similar acyl chain lengths, their headgroups vary drastically. As such, it is expected that the packing behavior of the different mixed lipid monolayers would be distinct from one another. This difference in packing behavior is quite evident from Figure 5 which displays the surface pressure-area isotherms of the various different lipid mixtures in the absence (Figure 5A) and presence of cholesterol (Figure 5B). As seen in Figure 5A, lipid combinations containing a greater percentage of POPE proceeds to a more condensed phase upon compression, with the mean area of ~67 Å^2^ and ~58 Å^2^ per molecule for POPE:POPC and POPE:POPS (5:1 mole ratio) monolayers respectively. In contrast, monolayers of POPC:POPE and POPS:POPE (where mole ratios have been reversed) show a mean area of ~96 Å^2^ and ~90 Å^2^ per molecule respectively. However, in the presence of cholesterol, the ordering of the mixed lipid monolayers by cholesterol is obvious from the condensing effect observed from the surface pressure-area isotherms in Figure 5B. Although cholesterol condenses the POPE:POPC and POPE:POPS monolayers to a similar mean area of ~60 Å^2^ per molecule, the insertion of CLIC1 within these monolayers were significantly different and may account for the presence of a completely different protein–lipid interaction. 

Several conventional studies of binary mixtures of two or more phospholipids have indicated that cholesterol may preferentially associate with different lipid classes in the same mixture [34]. Recent studies have shown clear evidence for the formation of cholesterol-poor or cholesterol-rich microdomains or clusters, which are also enriched or depleted of certain phospholipid classes, which occur in both model and biological membranes [34,35,36]. It was concluded that limiting amounts of cholesterol show comparable or higher affinities for anionic phospholipids than for zwitterionic phospholipids and the order of preference of cholesterol thus established was Sphingomyelin (SM) > Phosphatidylserine (PS) > Phosphatidylglycerol (PG) > Phosphatidylcholine (PC) > Phosphatidylethanolamine (PE) [34,35,36]. Hence, CLIC1 proteins show greater interaction in mixed monolayers containing anionic POPS and cholesterol (Figure 4). The fact that CLIC1 interacts with POPE not POPS (see Figure 2) and also that cholesterol will preferentially form microdomains with POPS over POPE, as a result CLIC1 protein shows greater interaction with POPE:POPS:Chol monolayer (mole ratio 4:1:1) which has a greater mole percent of POPE for CLIC1 interaction in comparison to POPS:POPE:Chol monolayer. It is also not surprising that CLIC1 shows greater levels of insertion to a POPC:POPE:Chol monolayer (ΔA ~9.3 ± 0.3) in comparison to POPE:POPC:Chol monolayer (ΔA ~4.8 ± 0.46%) (See Figure 4, black bars) for such an interaction was evident from the results shown in Figure 2 and Figure 3.

### 2.5. CLIC1–Cholesterol Interaction

Our previous studies using tBLMs, had shown that preincubation of CLIC1 protein with cholesterol diminishes ion channel activity of the protein [8]. This inhibition of CLIC1 ion channel activity by cholesterol could result either from a reduction in its membrane binding or an interference with the oligomerisation step within the target membrane. Previous studies with different CDCs have shown inconsistencies in the role of cholesterol in the toxins’ cytolytic mechanisms. Studies with streptolysin O have shown that the toxin requires cholesterol for the initial event of membrane binding and this binding can be inhibited by exogenous cholesterol or oxidation [37]. On the other hand, preincubation of listeriolysin O with cholesterol interfered with oligomerisation thus inhibiting its cytolytic activity but did not impair its membrane binding ability [38]. Hence, to further elucidate whether the interaction between CLIC1 and cholesterol was occurring pre- or post- CLIC1 insertion into the membrane, experiments were carried out which involved pre-incubation of CLIC1 with cholesterol prior to its addition to the POPC:Chol (5:1 mole ratio) monolayer. SDS-PAGE analysis of protein samples collected pre- and post- CLIC1 incubation with cholesterol (followed by high speed centrifugation) confirmed that the bulk of the protein remains soluble and thus, available for interaction with the lipid monolayer (results not shown). 

**Figure 6:**
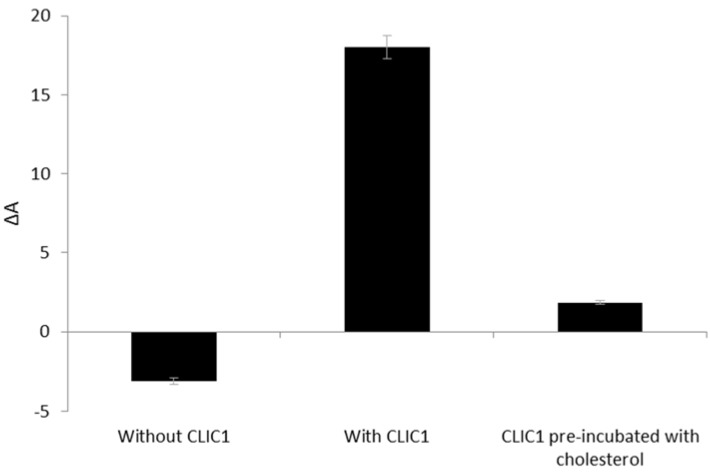
Percent area expansion profiles of POPC:Chol monolayer after 3 h without CLIC1 protein or after injection of recombinant CLIC1 wild-type and pre-incubated CLIC1 protein. CLIC1 was pre-incubated with cholesterol for an hour on ice prior to addition of the pre-incubated protein sample to the monolayer held at a constant surface pressure of 20 mN·m^−1^. Pre-incubation of CLIC1 protein with cholesterol resulted in complete abrogation of CLIC1 insertion into the monolayer.

Figure 6 shows the percent trough area expansion of POPC:Chol monolayer held at a constant surface pressure of 20 mN·m^−1^ after 3 h without CLIC1 and following insertion of recombinant CLIC1/pre-incubated CLIC1 into the subphase. As seen in Figure 6, pre-incubation of CLIC1 protein with cholesterol prior to its addition to the monolayer, significantly (*p* > 0.005) reduced the spontaneous membrane insertion of CLIC1 into POPC:Chol monolayer by 96 ± 0.5% in comparison to the insertion of non-incubated CLIC1 protein into the monolayer. These findings thereby suggests that pre-incubation of CLIC1 with cholesterol results in the formation of a relatively stable interaction (pre-complex) between the protein and cholesterol in the aqueous subphase which in turn prevents CLIC1 from inserting into the membrane. Therefore, it appears likely that in its soluble form CLIC1 may also interact with cytosolic cholesterol and this may in turn also regulate the proteins’ insertion into the membrane. Whether cholesterol is also needed for the proteins’ oligomerisation remains unclear but it seems clear that it is required and facilitates CLIC1 insertion/ binding to the membrane. The formation of a pre-complex with cholesterol also suggests that CLIC1 contains a cholesterol binding domain and when this is occupied by cholesterol the protein in solution no longer can bind to membrane cholesterol. Interestingly, analysis of amino acid sequences done previously by our group [8] have revealed a GXXXG motif at the N-terminal domain that is highly conserved amongst all the human CLIC proteins, several members of the CDCs (examples: listeriolysin, perfringolysin-O) [8] as well as several human membrane proteins (examples: glycophorin-A, ErbB receptor, G-protein coupled receptors etc.) [39]. Studies have shown that the GXXXG motif facilitates the homo or hetero-oligomerisation of membrane proteins [40] and acts as a cholesterol binding site in amyloid precursor proteins [6]. This GXXXG motif may well be the cholesterol binding site in the CLIC proteins and we are currently investigating the role of this motif in CLIC1’s interaction with cholesterol. 

## 3. Materials and Methods

Three lipids were used to evaluate the CLIC1–membrane interactions at the air–liquid interface: zwitterionic 1-palmitoyl-2-oleoyl-*sn*-glycero-3-phosphocholine (POPC) and 1-palmitoyl-2-oleoyl-sn-glycero-3-phosphoethanolamine (POPE) and anionic 1-palmitoyl-2-oleoyl-*sn*-glycero-3-phospho-L-serine (POPS). The lipids were used alone or in the following 5:1 mole ratio combinations with cholesterol (Chol)–POPC:Chol; POPS:Chol and POPE:Chol. Mixed lipid monolayers were formed using 5:1 mole ratio combinations of the different phospholipids or with cholesterol in a mole ratio of 4:1:1. All lipids were purchased from Avanti Polar Lipids (Alabaster, USA) and used as received. Cholesterol was purchased from Sigma Aldrich (Australia). Lipid stock solutions were prepared in spectroscopic grade chloroform (Sigma) at a concentration of 1 mg·mL^−1^ and stored at –20 °C. 

### 3.1. Protein Expression and Purification

Recombinant CLIC1 protein was expressed as 6xHis-CLIC1 fusion proteins in E.coli BL21 (DE3) cells (in 2x YT medium, induction with 1 mM IPTG (Sigma Aldrich) at 20 °C, overnight with shaking at ~200 rpm). rotein was purified as previouCells were harvested and the recombinant CLIC1 psly described [8], with the exception that a Hiprep 16/60 Sephacryl S-100 HR (GE Healthcare Life Sciences) size exclusion chromatography column was used for further purification. The purified protein is equilibrated in column sizing buffer (100 mM KCl, 20 mM HEPES, 1 mM NaN_3_, pH 7.5) containing 0.5 mM TCEP to maintain a reducing environment. Protein purity was determined by SDS-PAGE and western-blot analysis using a 12.5% acrylamide gel slab according to the method of Laemmli. All purified CLIC1 proteins comprised of the CLIC1 sequence and an additional three residues (Gly-Ser-His) at the N-terminus as a result of the thrombin cleavage site in the fusion construct.

### 3.2. Surface Activity of CLIC1 Protein at the Air–Water Interface

To evaluate the surface activity of CLIC1 protein, the change in surface pressure (π) by the adsorption of CLIC1 protein from a KCl/Hepes buffer (0.1M KCL, 0.1 mM HEPES and 0.01 mM CaCl_2_, pH 6.5) subphase was measured. The experiments were carried out in triplicate at room temperature (~25 °C) on a 25 mL KCl/Hepes buffer subphase using a computer controlled Langmuir trough (Nima Technologies, Coventry, UK). The rectangular trough had a surface area of 90 cm^2^ and the surface pressure (π) was measured by the Wilhelmy method using a filter paper plate. Prior to each experiment, the Langmuir trough was thoroughly cleaned with chloroform and rinsed with MilliQ water and then filled with 25 mL buffer solution and checked for cleanliness by running isotherm cycles. Before injecting CLIC1 into the buffer subphase, barriers were partially closed to give a total surface area of 50 cm^2^, roughly the same as the surface area of the lipid monolayers compressed to 20 mN·m^−1^ for the subsequent insertion experiments. To achieve a final CLIC1 concentration of 2 µg mL^−1^, 0.2 mL of 50 ug CLIC1 was injected into the subphase using a glass microsyringe.

### 3.3. Interaction of CLIC1 with Lipid Monolayers

To evaluate the interactions between CLIC1 and lipid membranes, insertion of CLIC1 into lipid monolayers held at a constant surface pressure (π) was measured. The lipid (/cholesterol) monolayers (mentioned above) were prepared by spreading dropwise 10 µL of the lipid stock solution onto the surface of the KCl/Hepes buffer (pH 6.5) in the Langmuir trough (Nima Technologies, Coventry, UK). For all Langmuir trough experiments, the volume of the aqueous subphase was kept constant at 25 mL and all experiments were carried out in triplicate and at room temperature (~25 °C). Once spread, the lipid monolayers were allowed to equilibrate for 5 min (to ensure complete evaporation of the chloroform) before isotherm cycles were performed to form a uniform monolayer. The barrier then symmetrically compressed the monolayer at 20 cm^2^·min^−1^ to a target π of 20 mN·m^−1^. The speed at which the barrier moves to maintain this target pressure was controlled by a feedback loop and depends upon how fast the barrier needs to expand or contract in response to a perturbation. Recombinant CLIC1 protein (50 µg) was then injected into the subphase underneath the lipid monolayer and changes in surface area were measured. The data is represented as percentage surface area expansion ΔA = [(A − A_i_)/A_i_] × 100, where A is the surface area at time t and A_i_ is the initial surface area of the monolayer when it reached 20 mN·m^−1^. Since the monolayer was kept at a constant π, the barrier expanded as a result of protein insertion and the percentage area expansion was taken as a measure of favourable CLIC1–lipid interactions. 

### 3.4. Pre-incubation of CLIC1 with Cholesterol

A total of 50 µL of the cholesterol stock solution (1 mg/mL) was added to 200 µL of HEPES/KCl buffer and the chloroform was removed using N_2_ gas for duration of one hour. Recombinant CLIC1 protein (50 µg) was then added to the buffer solution containing the cholesterol and incubated for an hour on ice prior to addition of this pre-incubated protein sample to the corresponding POPC:Chol monolayer held at a constant π of 20 mN·m^−1^ in the Langmuir trough. CLIC1-wt samples were analysed by SDS-PAGE before and after incubation with cholesterol followed by centrifugation at 14,000 rpm for 15 min. As a control, CLIC1-wt protein was pre-incubated in buffer only without cholesterol and samples were collected as mentioned above. 

## 4. Conclusions

Our results provide insight into the regulatory role that phospholipids and cholesterol play in the spontaneous membrane insertion of the protein CLIC1. Although, there are variations in CLIC1 interaction with different phospholipid monolayers, it is however clear that CLIC1 has a strong preference for associating with or intercalating into pure lipid or mixed lipid monolayers containing cholesterol. The Langmuir monolayer film experiments reported here show that the optimal membrane composition for CLIC1 insertion is a combination of POPE, POPS and cholesterol in a mole ratio of 4:1:1. Since cholesterol has been shown to induce insertion of several membrane proteins, it seems that cholesterol also renders the otherwise soluble and stable cytosolic CLIC1 for optimal membrane insertion. Furthermore, CLIC1’s apparent ability to form a relatively stable pre-complex with cholesterol suggests the presence of a cholesterol binding domain in CLIC1. Our results in the current study complement our previously published study using tBLMs. This study supports the model that CLIC1 binds to cholesterol in the membrane via a putative cholesterol-binding domain for its initial docking onto the membrane. It then inserts into phospholipid monolayers with varying kinetics dependent upon the lipid profile. Subsequent oligomerisation of CLIC1 protein monomers in the membrane would then likely occur, in order for functional ion channels to be formed. 

## Abbreviations

POPC: 1-palmitoyl-2-oleoylphosphatidylcholine; POPE: 1-palmitoyl-2-oleoyl-sn-glycero-3-phospho-ethanolamine; POPS: 1-palmitoyl-2-oleoyl-*sn*-glycero-3-phospho-L-serine; Chol: cholesterol; tBLMs: tethered bilayer membranes; CDCs: cholesterol-dependent cytolysins.

## References

[1] Liscum L., Underwood K. (1995). Intracellular cholesterol transport and compartmentation. J. Biol. Chem..

[2] Simons K., Ikonen E. (2000). How cells handle cholesterol. Science.

[3] Mouritsen O., Zuckermann M. (2004). What’s so special about cholesterol?. Lipids.

[4] Gilbert R. (2010). Cholesterol-dependent cytolysins. Adv. Exp. Med. Biol..

[5] Epand R. (2006). Cholesterol and the interaction of proteins with membrane domains. Prog. Lipid Res..

[6] Barrett P., Song Y., Van Horn W., Hustedt E., Schafer J., Hadziselimovic A., Beel A., Sanders C. (2012). The amyloid precursor protein has a flexible transmembrane domain and binds cholesterol. Science.

[7] Yu X., Zheng J. (2012). Cholesterol promotes the interaction of alzheimer β-amyloid monomer with lipid bilayer. J. Mol. Biol..

[8] Valenzuela S., Alkhamici H., Brown L., Almond O., Goodchild S., Carne S., Curmi P., Holt S., Cornell B. (2013). Regulation of the membrane insertion and conductance activity of the metamorphic chloride intracellular channel protein clic1 by cholesterol. PLoS ONE.

[9] Harrop S., DeMaere M., Fairlie W., Reztsova T., Valenzuela S., Mazzanti M., Tonini R., Qiu M., Jankova L., Warton K. (2001). Crystal structure of a soluble form of the intracellular chloride ion channel clic1 (ncc27) at 1.4-a resolution. J. Biol. Chem..

[10] Valenzuela S., Martin D., Por S., Robbins J., Warton K., Bootcov M., Schofield P., Campbell T., Breit S. (1997). Molecular cloning and expression of a chloride ion channel of cell nuclei. J. Biol. Chem..

[11] Littler D., Harrop S., Goodchild S., Phang J., Mynott A., Jiang L., Valenzuela S., Mazzanti M., Brown L., Breit S. (2010). The enigma of the clic proteins: Ion channels, redox proteins, enzymes, scaffolding proteins?. FEBS Lett..

[12] Tulk B., Kapadia S., Edwards J. (2002). Clic1 inserts from the aqueous phase into phospholipid membranes, where it functions as an anion channel. Am. J. Physiol. Cell Physiol..

[13] Warton K., Tonini R., Fairlie W., Matthews J., Valenzuela S., Qiu M., Wu W., Pankhurst S., Bauskin A., Harrop S. (2002). Recombinant clic1 (ncc27) assembles in lipid bilayers via a ph-dependent two-state process to form chloride ion channels with identical characteristics to those observed in chinese hamster ovary cells expressing clic1. J. Biol. Chem..

[14] Tulk B., Schlesinger P., Kapadia S., Edwards J. (2000). Clic-1 functions as a chloride channel when expressed and purified from bacteria. J. Biol. Chem..

[15] Valenzuela S., Mazzanti M., Tonini R., Qiu M., Warton K., Musgrove E., Campbell T., Breit S. (2000). The nuclear chloride ion channel ncc27 is involved in regulation of the cell cycle. J. Physiol..

[16] Novarino G., Fabrizi C., Tonini R., Denti M., Malchiodi-Albedi F., Lauro G., Sacchetti B., Paradisi S., Ferroni A., Curmi P. (2004). Involvement of the intracellular ion channel clic1 in microglia-mediated beta-amyloid-induced neurotoxicity. J. Neurosci..

[17] Al Khamici H., Brown L., Hossain K., Hudson A., Sinclair-Burton A., Ng J., Daniel E., Hare J., Cornell B., Curmi P. (2015). Members of the chloride intracellular ion channel protein family demonstrate glutaredoxin-like enzymatic activity. PLoS ONE.

[18] Goodchild S., Howell M., Littler D., Mandyam R., Sale K., Mazzanti M., Breit S., Curmi P., Brown L. (2010). Metamorphic response of the clic1 chloride intracellular ion channel protein upon membrane interaction. Biochemistry.

[19] Littler D., Harrop S., Fairlie W., Brown L., Pankhurst G., Pankhurst S., DeMaere M., Campbell T., Bauskin A., Tonini R. (2004). The intracellular chloride ion channel protein clic1 undergoes a redox-controlled structural transition. J. Biol. Chem..

[20] Goodchild S., Howell M., Cordina N., Littler D., Breit S., Curmi P., Brown L. (2009). Oxidation promotes insertion of the clic1 chloride intracellular channel into the membrane. Eur. Biophys. J..

[21] Singh H., Ashley R. (2006). Redox regulation of clic1 by cysteine residues associated with the putative channel pore. Biophys. J..

[22] Fanucchi S., Adamson R., Dirr H. (2008). Formation of an unfolding intermediate state of soluble chloride intracellular channel protein clic1 at acidic ph. Biochemistry.

[23] Cromer B., Gorman M., Hansen G., Adams J., Coggan M., Littler D., Brown L., Mazzanti M., Breit S., Curmi P. (2007). Structure of the janus protein human clic2. J. Mol. Biol..

[24] Littler D., Assaad N., Harrop S., Brown L., Pankhurst G., Luciani P., Aguilar M., Mazzanti M., Berryman M., Breit S. (2005). Crystal structure of the soluble form of the redox-regulated chloride ion channel protein clic4. FEBS J..

[25] Littler D., Harrop S., Brown L., Pankhurst G., Mynott A., Luciani P., Mandyam R., Mazzanti M., Tanda S., Berryman M. (2008). Comparison of vertebrate and invertebrate clic proteins: The crystal structures of caenorhabditis elegans exc-4 and drosophila melanogaster dmclic. Proteins.

[26] Stoychev S., Nathaniel C., Fanucchi S., Brock M., Li S., Asmus K., Woods V.J., Dirr H. (2009). Structural dynamics of soluble chloride intracellular channel protein clic1 examined by amide hydrogen-deuterium exchange mass spectrometry. Biochemistry.

[27] García-Sáez A., Mingarro I., Pérez-Payá E., Salgado J. (2004). Membrane-insertion fragments of bcl-xl, bax, and bid. Biochemistry.

[28] Tweten R. (2005). Cholesterol-dependent cytolysins, a family of versatile pore-forming toxins. Infect. Immun..

[29] Ege C., Lee K. (2004). Insertion of alzheimer’s a beta 40 peptide into lipid monolayers. Biophys. J..

[30] Hauser H., Pascher I., Pearson R., Sundell S. (1981). Preferred conformation and molecular packing of phosphatidylethanolamine and phosphatidylcholine. Biochim. Biophys. Acta.

[31] Dill K., Stigter D. (1988). Lateral interactions among phosphatidylcholine and phosphatidylethanolamine head groups in phospholipid monolayers and bilayers. Biochemistry.

[32] Domènech O., Torrent-Burgués J., Merino S., Sanz F., Montero M., Hernández-Borrell J. (2005). Surface thermodynamics study of monolayers formed with heteroacid phospholipids of biological interest. Colloids Surf. B Biointerfaces.

[33] Langner M., Kubica K. (1999). The electrostatics of lipid surfaces. Chem. Phys. Lipids.

[34] Demel R., Jansen J., van Dijck P., van Deenen L. (1977). The preferential interaction of cholesterol with different classes of phospholipids. Biochim. Biophys. Acta.

[35] McMullen T., Lewis R., McElhaney R. (2000). Differential scanning calorimetric and fourier transform infrared spectroscopic studies of the effects of cholesterol on the thermotropic phase behavior and organization of a homologous series of linear saturated phosphatidylserine bilayer membranes. Biophys. J..

[36] Van Dijck P., De Kruijff B., Van Deenen L., De Gier J., Demel R. (1976). The preference of cholesterol for phosphatidylcholine in mixed phosphatidylcholine-phosphatidylethanolamine bilayers. Biochim. Biophys. Acta.

[37] Alouf J., Geoffroy C., Pattus F., Verger R. (1984). Surface properties of bacterial sulfhydryl-activated cytolytic toxins. Interaction with monomolecular films of phosphatidylcholine and various sterols. Eur. J. Biochem..

[38] Jacobs T., Darji A., Frahm N., Rohde M., Wehland J., Chakraborty T., Weiss S. (1998). Listeriolysin o: Cholesterol inhibits cytolysis but not binding to cellular membranes. Mol. Microbiol..

[39] Prakash A., Janosi L., Doxastakis M. (2011). Gxxxg motifs, phenylalanine, and cholesterol guide the self-association of transmembrane domains of erbb2 receptors. Biophys. J..

[40] Senes A., Engel D., DeGrado W. (2004). Folding of helical membrane proteins: The role of polar, gxxxg-like and proline motifs. Curr. Opin. Struct. Biol..

